# Hallmarks and Molecular Tools for the Study of Mitophagy in Parkinson’s Disease

**DOI:** 10.3390/cells11132097

**Published:** 2022-07-02

**Authors:** Thomas Goiran, Mohamed A. Eldeeb, Cornelia E. Zorca, Edward A. Fon

**Affiliations:** McGill Parkinson Program, Neurodegenerative Diseases Group, Department of Neurology and Neurosurgery, Montreal Neurological Institute, McGill University, Montreal, QC H3A 2B4, Canada; thomas.goiran@mail.mcgill.ca (T.G.); mohamed.eldeeb@mcgill.ca (M.A.E.); cornelia.zorca@mcgill.ca (C.E.Z.)

**Keywords:** protein quality control, Parkinson’s disease, mitochondrial quality control, ubiquitin, alpha-syn, mitophagy, PINK1, Parkin, mito-Keima, mito-QC, mito-SRAI

## Abstract

The best-known hallmarks of Parkinson’s disease (PD) are the motor deficits that result from the degeneration of dopaminergic neurons in the substantia nigra. Dopaminergic neurons are thought to be particularly susceptible to mitochondrial dysfunction. As such, for their survival, they rely on the elaborate quality control mechanisms that have evolved in mammalian cells to monitor mitochondrial function and eliminate dysfunctional mitochondria. Mitophagy is a specialized type of autophagy that mediates the selective removal of damaged mitochondria from cells, with the net effect of dampening the toxicity arising from these dysfunctional organelles. Despite an increasing understanding of the molecular mechanisms that regulate the removal of damaged mitochondria, the detailed molecular link to PD pathophysiology is still not entirely clear. Herein, we review the fundamental molecular pathways involved in PINK1/Parkin-mediated and receptor-mediated mitophagy, the evidence for the dysfunction of these pathways in PD, and recently-developed state-of-the art assays for measuring mitophagy in vitro and in vivo.

## 1. Introduction

Mitochondria are essential organelles that possess their own genome and provide energy in the form of ATP for a variety of cellular processes [1,2,3,4]. For energy production, mitochondria rely on the process of oxidative phosphorylation (OXPHOS). The components of the OXPHOS machinery are encoded both in the nuclear and mitochondrial DNA. Dysfunction of OXPHOS components, especially of complex I, have been implicated with Parkinson’s disease (PD), among other neurodegenerative diseases [5]. Early evidence of this came from observations that two complex I inhibitors, MPTP and rotenone, cause death of PD-associated dopamine neurons in both humans and rodent models [6,7]. Dysfunctional or otherwise damaged mitochondria are cleared by a specialized form of macroautophagy, called mitochondrial autophagy or mitophagy. A subset of sporadic forms of PD are thought to be associated with impaired mitochondrial function, though whether complex I defects are a cause or consequence of factors such as oxidative stress, is currently unclear. Likewise, familial forms of PD have been traced to mutations in genes encoding proteins associated with mitochondrial function and mitophagy, such as PINK1 and Parkin [8,9]. Moreover, in addition to genetic factors, environmental factors that affect mitochondria are also thought to play key roles in PD pathogenesis [10,11,12].

**Figure 1:**
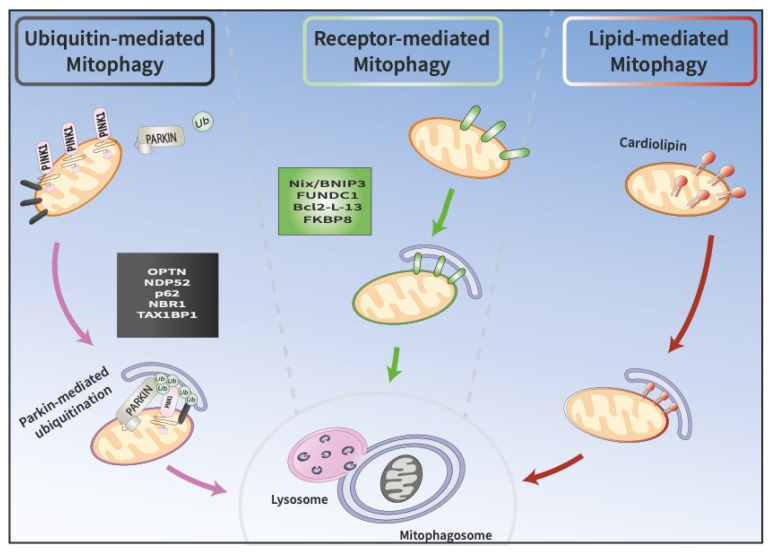
Three pathways at the crossroads of mitophagy. A. Ubiquitin-mediated mitophagy involves the recruitment of PINK1 and Parkin to the OMM, which promote the sequestration of damaged mitochondria into phagophores called mitophagosomes. Mitophagosomes subsequently fuse with lysosomes, where cargo is degraded. B. Receptor-mediated mitophagy depends on the direct binding of unique receptors, such as NIX/BNIP3L or FUNCD1, to LC3 on autophagosomes, which target damaged mitochondria for degradation. C. In lipid-mediated mitophagy, cardiolipin is externalized from the IMM to the OMM, where it binds to LC3 on mitophagosomes.

Cells possess several non-redundant mitophagy pathways; each can be triggered in response to different stimuli and each can elicit mitophagy through the activation of distinct signaling cascades (Figure 1) [13]. For instance, PINK1/Parkin dependent mitophagy is the main modulator of turnover of depolarized mitochondria. Additionally, several mitochondrial proteins, such as BNIP3, NIX, PHB2, and FUNDC1, have been shown to function as mitophagy receptors. These receptors are localized at the outer mitochondrial membrane (OMM) and interact directly with the autophagosomal membrane protein light chain 3 (LC3) to stimulate mitophagy. In addition, lipid-mediated mitophagy and ubiquitin-mediated mitophagy have also been reported [14,15,16]. Collectively, these pathways are deregulated in many human diseases, including neurodegenerative disorders, aging, and cancer [8,17,18,19,20]. In this review, we provide an overview of the key pathways involved in the regulation of mitophagy and their association with PD. We also discuss the molecular toolbox currently available to study this process in vitro and in vivo.

## 2. Molecular Pathways of Mitophagy

### 2.1. PINK1/Parkin-Mediated Ubiquitin-Driven Mitophagy

**Figure 2:**
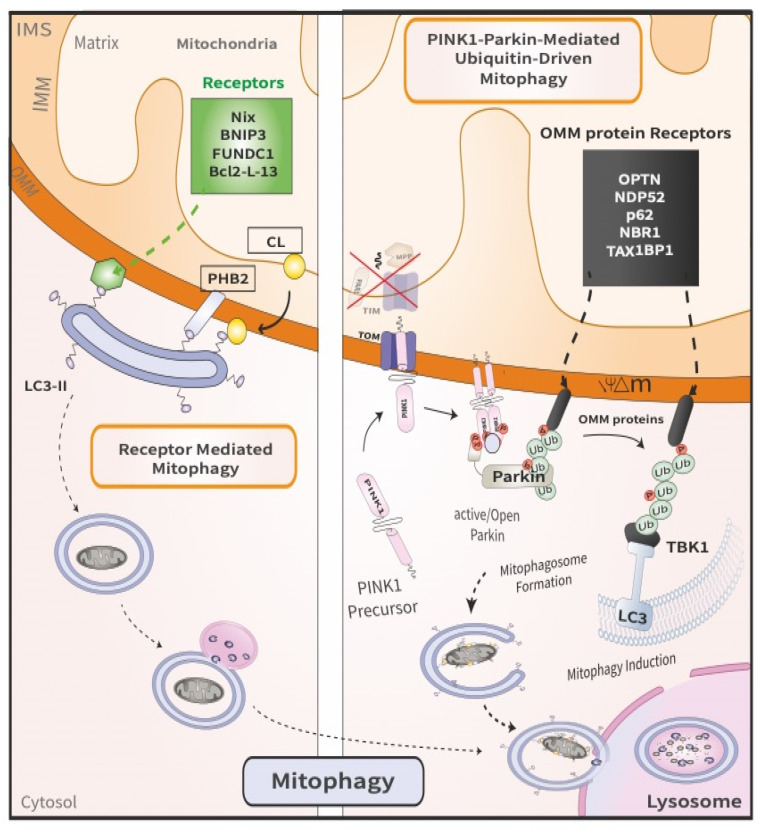
Comparison between PINK1/Parkin-mediated mitophagy and receptor-mediated mitophagy. The latter (**left**) involves the direct binding of mitophagy receptors to LC3 on the autophagosomes, which then deliver the engulfed damaged mitochondria to the lysosome. By contrast, the former (**right**) is a multi-step process that ensues following the loss of mitochondrial membrane potential. First, PINK1 is stabilized on the OMM of damaged mitochondria. Following dimerization, PINK1 recruits and phosphorylates Parkin, thereby initiating mitophagy.

Ubiquitin (Ub) is a highly conserved small protein of 76 amino acids that is present in all eukaryotic cells. Ub plays a crucial role in many cellular processes, including protein degradation and immune system signaling. Ubiquitin-dependent protein degradation involves an enzymatic cascade resulting in the covalent conjugation of ubiquitin to the target protein substrate. This multi-step biochemical cascade leads either to the targeted degradation or to the altered localization of the substrate. The ubiquitination process is carried out by three enzymes: E1 (the ubiquitin activating enzyme), E2 (the ubiquitin conjugating enzyme, or carrier enzyme), and E3 (the ubiquitin protein-ligase). E3 Ub ligases are the principal factors that determine substrate specificity and are essential players in the ubiquitin pathway [21]. Parkin (encoded by the *PRKN* or *PARK2* gene) is an E3 Ub ligase, discovered in 1998, implicated in the pathogenesis of autosomal recessive PD (ARPD) [22,23,24]. Parkin contains five domains: an N-terminal Ub-like domain (UBL), a RING1 domain, an IBR domain, a RING2 domain, and a RING0 domain that is unique to Parkin [25,26,27]. Another ARPD-associated gene, PINK1 (PTEN-induced putative kinase 1), which is encoded by the *PARK6* gene, was discovered in 2001 [8,28] and encodes a mitochondrial serine/threonine kinase that regulates Parkin activity via phosphorylation. The PINK1 protein contains different domains, including an N-terminal mitochondrial targeting sequence (MTS), a transmembrane domain (TMD) followed by a serine/threonine kinase domain, and a regulatory domain at the C-terminus [29]. Under steady-state conditions, Parkin is located in the cytosol and is in an autoinhibited state. Concurrently, PINK1 is maintained at a low-level, owing to mitochondrial import, protease cleavage, and proteasomal degradation. Indeed, PINK1 is imported by the translocase of the outer membrane (TOM) complex into the inter membrane space (IMS) and the mitochondrial inner membrane (MIM), and then cleaved by the matrix processing peptidase (MPP) and the presenilin-associated rhomboid (PARL) in the N-terminal portion between Ala103 and Phe104. It is then retro-translocated to the cytosol, where the newly generated N-terminus, now consisting of the destabilizing amino acid Phe104, is constitutively recognized by N-end rule E3 ubiquitin ligases (UBR1, UBR2, and UBR4), leading to degradation of PINK1 by the proteasome [30,31,32,33,34]. However, reduction in the mitochondrial membrane potential results in the failure of PINK1 import and its accumulation on the outer mitochondrial membrane (OMM). This in turn leads to PINK1 dimerization and autophosphorylation at Ser228 and Ser402, resulting in its activation [35,36,37,38]. Thus, PINK1 accumulation on the OMM functions as a mitochondrial damage sensor that, once activated, triggers mitophagy and mediates downstream phosphorylation events, including the phosphorylation of ubiquitin at Ser65 and the phosphorylation of Ser65 in the UBL domain of Parkin. Phospho-ubiquitin (pSer65-Ub), is conjugated to proteins on the outer mitochondrial membrane; it then serves as a receptor for Parkin recruitment from the cytosol to mitochondria [36,39,40,41,42], and contributes to fully activating Parkin by inducing conformational changes in the Parkin core and releasing the UBL domain [43,44,45,46,47,48,49]. The precise mechanism by which Ubl phosphorylation activates Parkin is complex in nature, resulting in a number of Parkin activation models [43,44,45,46,47,48,49]. Subsequently, activated Parkin conjugates additional Ub moieties onto OMM proteins, marking the mitochondria for degradation by the autophagic machinery [18], thereby triggering mitophagy. Upon activation, Parkin polyubiquitinates several proteins on the OMM, including MFN1/2, TOM20/40/70, and VDAC 1 [50,51]. MFN1/2 (mitofusin), two GTPases required for mitochondrial fusion, were among the first and most crucial targets of Parkin-mediated ubiquitination. Mitochondria become progressively fragmented as a result of proteasomal degradation of MFN, resulting in their separation from one another. This phase appears to be crucial in distinguishing damaged mitochondrial fragments from the healthy reticulum that remains [49,50]. The ubiquitination of OMM proteins also facilitates the recruitment of receptor proteins that are part of the downstream autophagic degradation machinery (mitophagy). On the one hand, receptor proteins, such as p62, interact directly with polyubiquitin chains, and on the other hand, with LC3s or GABARAPs [51]. Initially, p62 was identified as the main adapter for PINK1/Parkin-mediated mitophagy [50]. Additional comprehensive studies identified five receptors: TAX1BP1, NDP52, NBR1, p62 and OPTN (Figure 2). Among these, NDP52 and OPTN were found to be the most important receptors for PINK1/Parkin-dependent mitophagy [52]. The recruitment of autophagy receptors, such as NDP52 and OPTN, to damaged mitochondria is a TANK-binding kinase 1- (TBK1) dependent process [52,53,54]. TBK1 is a serine/threonine kinase that enhances the binding ability of autophagy receptors to various Ub chains through their phosphorylation [52,53,54]. In the presence of PINK1 and Parkin, TBK1 activation also requires OPTN binding to Ub chains [53,54]. In the current mitophagy model, OPTN and NDP52 recruit phagophores to mitochondria by directly binding to LC3 through their LC3-interacting region (LIR), after binding to polyubiquitin chains [55,56]. A previous study has highlighted the role of NDP52 in the recruitment of the ULK1 complex to damaged mitochondria [57]. NDP52 directly interacts with FIP200 in a TBK1-dependent manner to recruit the ULK1 complex, leading to autophagosome biogenesis on damaged mitochondria and to the recruitment of the autophagy machinery. Therefore, receptor proteins ensure the removal of mitochondria by autophagosomes downstream of PINK1/Parkin signaling.

### 2.2. Receptor Mediated Mitophagy

Several mitophagy receptors, such as ATG32 in yeast [58], as well as BNIP3 (BCL2 and adenovirus E1B 19-kDa-interacting protein 3) [59], NIX (also known as BNIP3L) [60], and FUNDC1 in mammalian cells, have been identified. One major characteristic of mitophagy receptors is that they contain LIR motifs that interact with LC3, thereby enhancing mitochondrial sequestration into phagophores [61,62,63,64]. The mechanism of BNIP3- and NIX-mediated mitophagy is distinct from that of the PINK1/Parkin pathway in that these proteins act as direct adaptors targeting mitochondria to the autophagosome. BNIP3 (a member of the pro-death BCL2 family of proteins) [65] and NIX (a homolog of BNIP3 with ~56% sequence similarity) [66] have a BH3 domain and a C-terminal transmembrane domain (TMD), which is crucial for their proapoptotic functions and mitochondrial localization [67,68]. Furthermore, BNIP3 and NIX have similar N-terminal LIR domains exposed to the cytosol that facilitates LC3s (microtubule-associated protein 1A/1B light chain) interactions for both receptors, or to GABARAP (gamma aminobutyric acid receptor-associated protein) for NIX, leading to the recruitment of autophagosomes and to the induction of mitophagy [61,69,70]. In these stress response pathways, the expression of BNIP3 is transcriptionally regulated by HIF−1, PPARγ, Rb/E2F, FoxO3, activated Ras, and p53, whereas that of NIX is regulated by HIF−1 and p53 [71,72,73]. Although BNIP3 and NIX are predominantly under transcriptional control, they are post-translationally modified for their mitophagic activity. Notably, it has been shown that serine phosphorylation at positions 17 and 24 adjacent to the LIR of BNIP3 and at positions 34 and 35 in the LIR domain of NIX enhances the interaction of these receptors with LC3, augmenting mitophagy [74].

Numerous lines of research suggest a possible crosstalk between the BNIP3/NIX receptor-mediated pathway and the PINK1/Parkin-mediated axis [75,76]. Specifically, NIX was implicated in PINK1/Parkin-mediated mitophagy as a ubiquitination substrate of Parkin that recruits NBR1 to the mitochondria [77]. Additionally, BNIP3-induced mitophagy is reduced in Parkin-deficient cells [78], and BNIP3 can stabilize PINK1 on the OMM, inhibiting its proteolytic degradation [79]. These results indicate that these pathways could cooperate with each other and may be partially redundant under particular cellular stress conditions to ensure effective mitophagy.

Another receptor-mediated mitophagy pathway hinges on the FUN14 domain containing 1 (FUNDC1). FUNDC1, an integral mitochondrial outer-membrane protein, is another important receptor for hypoxia-mediated mitophagy. FUNDC1 is composed of three transmembrane domains (TMDs) and an LIR domain in its N-terminus exposed to the cytosol, which interacts with LC3 for autophagosome recruitment [80]. Like other key regulators of mitophagy, the activity of FUNDC1 is also regulated by cycles of phosphorylation and dephosphorylation. The phosphorylation states of the three key residues, Ser13, Ser17 and Tyr18, in the outer membrane region of FUNDC1, play essential roles in fine-tuning the binding affinity for LC3 and controlling mitophagy [81,82]. Under steady-state conditions, the LIR motif of FUNDC1 is phosphorylated at Ser13 by CSNK2/CK2 kinase and at Tyr18 by SRC kinase, which leads to inhibition of its interaction with LC3 and prevents mitophagy. Conversely, hypoxia elicits dephosphorylation of FUNDC1, which can then bind to LC3 and elicit mitophagy [82]. Besides hypoxia, the array of cellular signals or states that can trigger receptor-mediated mitophagy remains to be fully elucidated [83,84,85,86,87,88,89,90,91,92].

## 3. Mitochondrial Defects in PD

### 3.1. Environmental Toxins as Risk Factors for PD

Among the mitochondrial defects associated with PD, reduced complex I activity has been found not only in the substantia nigra [83,84,85], but also in various other cells and tissues, including fibroblasts, lymphocytes, platelets, and in the skeletal muscle of sporadic PD patients [86,87,88,89,90,91,92,93,94,95]. However, mitochondrial complex I inhibition was shown to harm dopaminergic neurons more than other types of neurons [96]. The conditional ablation of an essential subunit of mitochondrial complex I, Ndufs2, in mouse dopaminergic neurons was recently shown to cause OXPHOS dysfunction and parkinsonian motor learning deficits that could be rescued by systemic levodopa administration [5]. Evidence of toxin-induced mitochondrial dysfunction has been recognized for over 30 years as a potential mechanism of dopaminergic neuronal loss associated with PD. Accidental exposure to MPTP (1-methyl−4-phenyl−1,2,3,6-tetrahydropyridine), a contaminant from the synthesis of MPPP (1-methyl−4-phenyl−4-propionoxy-piperidine), has been correlated with the rapid onset of parkinsonism [97]. Notably, MPTP itself is not toxic, but MPP+, its oxidized form, becomes toxic after being metabolized by mitochondrial monoamine oxidase B (MAO-B). MPP+ is selectively taken up into DA neurons through the dopamine transporter (DAT). Once internalized into neurons, MPP+ is rapidly concentrated in mitochondria [98,99,100], blocking electron transfer at complex I [101]. Such blockade results in the suppression of the complex I-mediated oxidation of nicotinamide adenine dinucleotide (NAD) and in OXPHOS dysfunction [102,103,104], thereby generating an abundance of free radicals (ROS), which has been proposed to contribute to DA neurodegeneration. Numerous studies have demonstrated that exposure to MPTP results in increased ROS levels, inhibition of mitochondrial respiration, DA neuron loss, and even cytoplasmic inclusions that share the characteristics of Lewy bodies (LB), the pathological hallmark of PD [105,106,107,108,109,110,111]. Interestingly, MPTP-treated mice that exhibited motor deficits and loss of TH expression in the substantia nigra, could be rescued by the co-administration of cell-permeable recombinant human Parkin [112]. Likewise, bypassing complex I and directly supplying the mitochondrial electron transport chain with complex II substrates enhanced OXPHOS and concomitantly reduced DA neurodegeneration in MPTP-treated mice [113]. Moreover, inhibition of complex I following MPTP treatment was shown to result in the degradation of the mitophagy receptor BNIP3L, in decreased protein ubiquitination, and in p62 inactivation [114,115,116], suggesting that impairments in both the ubiquitin-proteasome system and in the autophagic pathway can accompany mitophagy defects, in this context.

Other complex I inhibitors implicated in PD pathophysiology include rotenone and paraquat [117,118,119]. Rotenone is a lipophilic compound capable of crossing the blood-brain barrier, as well as cellular membranes. Like MPP+, rotenone inhibits complex I of the mitochondrial electron transport chain, resulting in increased ROS production, decreased ATP synthesis, and apoptotic cell death [117,120]. Increased ROS levels lead to mitochondrial dysfunction correlated with dopaminergic neuronal death [117,120]. In vivo proteomics studies have analyzed alterations in the striatum and substantia nigra caused by rotenone treatment [117,120]. Notably, the majority of altered proteins identified in these studies were involved in dopamine signaling, calcium signaling, apoptosis, and mitochondrial maintenance. Exposure to most of these PD environmental contaminants results in increased cellular ROS levels, inhibition of mitochondrial respiration, DA neuron loss, and LB-like inclusions [117,121,122].

### 3.2. Genetic Links to PD Risk

Over the past three decades, genetic studies have identified both dominantly and recessively inherited genes associated with familial forms of PD. Examples of the former include *SNCA* (*PARK1*) and *LRRK2* (*PARK8*), while examples of the latter include *PINK1*, and *PRKN*. Among these, *SNCA* and *LRRK2* have recently been associated with deficient mitochondrial function and homeostasis linked to PD pathophysiology. Specifically, mitochondrial α-synuclein accumulation was observed in a variety of neuronal and animal models, as well as in postmortem brain tissue of patients suffering from PD [95,123]. One way α-synuclein is thought to cause mitochondrial dysfunction is by binding to the translocase of the outer mitochondrial membrane 20 (TOMM20) and by inhibiting mitochondrial protein import [124]. Additionally, α-synuclein can directly inhibit complex I, complex IV, and ATP synthase, resulting in altered mitochondrial respiration and in mtDNA damage [125,126,127,128,129]. Recent evidence has implicated another familial PD gene, LRRK2, in the clearance of dysfunctional mitochondria. Hsieh et al. demonstrated that the pathogenic G2019S LRRK2 variant slowed the initiation of mitophagy in iPSC-derived neurons through a mechanism involving the delayed removal of a mitochondrial outer membrane protein, Miro1 [130]. Corroborating this, Singh et al. found that the hyperactive G2019S LRRK2 variant exhibited reduced mitophagy in dopaminergic neurons and microglia, which could be pharmacologically rescued by treatment with the GSK3357679A kinase inhibitor [131]. Lastly, increased levels of mitochondrial DNA (mtDNA) have been detected in the cerebrospinal fluid (CSF) of symptomatic G2019S LRRK2 carriers compared to asymptomatic carriers of this mutation [132]. While these findings identified mtDNA as a potential biomarker for LRRK2-associated PD, the link between circulating, cell-free mtDNA and mitochondrial dysfunction remains unclear [133].

Within the recessive category of genes, *PINK1* and *PRKN* have been shown to be directly involved in sensing and removing damaged mitochondria as described in the previous sections. Mutations in *PINK1* and *PRKN* have been associated with PD in different model systems. In Drosophila melanogaster, Caenorhabditis elegans, and Danio rerio (zebrafish), *PINK1* loss leads to anomalies in mitochondrial morphology and function, including decreased ATP production and increased ROS, as well as in neurodegeneration and locomotor deficits [134,135,136]. Germline *PINK1* knockout and *PRKN* knockout mice, on the other hand, show mitochondrial malfunction and increased sensitivity to oxidative stress, accompanied by minimal PD-like pathology [137,138,139]. However, upon aging, *PRKN* knockout mice were found to have both motor dysfunction and TH neuronal loss in the substantia nigra that correlated with the accumulation of damaged mitochondria within the dopaminergic neurons [140]. Likewise, *PRKN* knockout mice expressing a proofreading-defective DNA polymerase γ (POLG), which rapidly accumulate mtDNA mutations, exhibited mitochondrial abnormalities and dopaminergic neuronal loss [141,142]. Different rat models of PD were also found to recapitulate diverse pathological hallmarks, including mitochondrial dysfunction manifested by altered expression levels of complex I subunits in the striatum, deficits in complex I-driven respiration [143], and elevated levels of oxidative damage [144]. Lastly, midbrain dopaminergic neurons derived from induced pluripotent stem cells (iPSCs) harboring mutations in the *PINK1* or *PRKN* loci, exhibited both abnormal mitochondrial morphology and decreased survival upon mitochondrial stress induction with carbonyl cyanide m-chlorophenyl hydrazone (CCCP) [145]. In summary, these studies highlight the important roles of *PINK1* and *PRKN* in regulating mitochondrial function associated with PD pathogenesis.

In addition to familial studies, recent genome wide association studies (GWAS) have identified 90 genes as risk factors for PD [146,147,148,149,150,151]. While some of the loci implicated in monogenic familial PD have been shown to act directly in mitochondrial quality control and to play key roles in mitophagy, other GWAS genes are thought to exert indirect effects, most prominently affecting autophagy and lysosomal function [152,153,154,155]. For example, Inositol−1,4,5-triphosphate (IP3) kinase B (*ITPKB*) was shown to modulate mitochondrial ATP production through calcium released from the ER [156]. To complement the GWAS, single-cell transcriptomic analyses of different populations within the substantia nigra and cortex identified cell-specific gene networks associated with PD in post-mortem brain samples [157]. Prominent among these networks were groups of genes involved in mitochondrial organization, oxidative phosphorylation, and the electron transport chain [157]. Taken together, these unbiased studies demonstrate how gene alterations affect mitochondrial function in PD.

## 4. Mitophagy Assays

**Figure 3:**
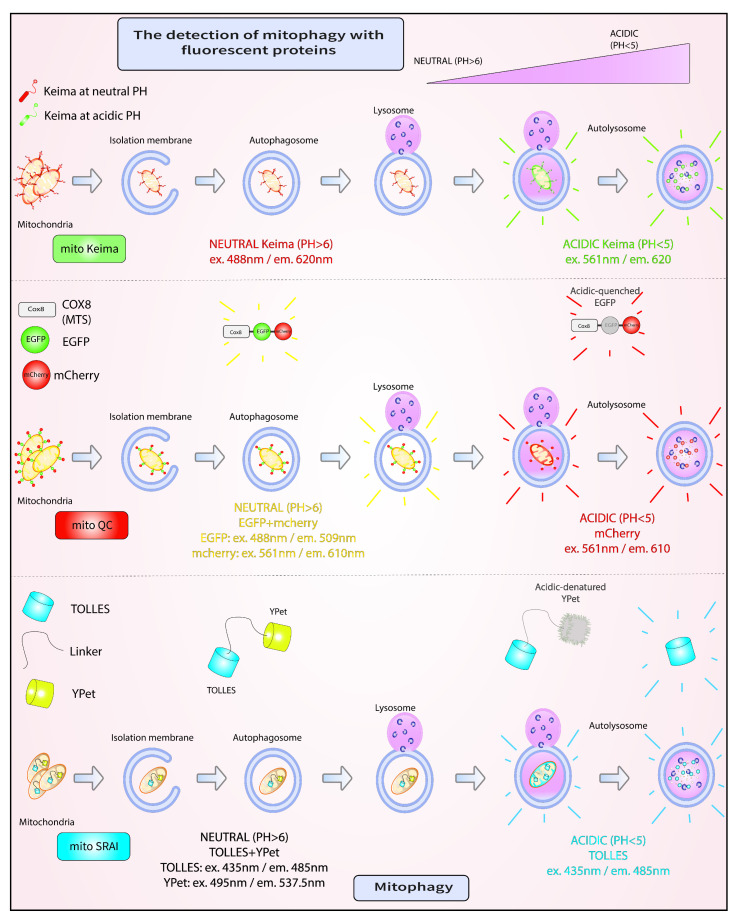
Fluorescent assays for mitophagy. A. mito-Keima is a pH-sensitive fluorescent biosensor, which fluoresces green at neutral pH in the cytosol, and red upon entry into acidic autolysosomes. B. mito-QC comprises two mitochondrially-targeted tandem fluorescent proteins, EGFP and mCherry. Both EGFP and mCherry fluoresce in the cytosol. However, in the lysosome, the fluorescence of mCherry is retained, while that of GFP is lost. C. mito-SRAI consists of two mitochondrially-targeted tandem fluorescent proteins, TOLLES and YPet. Unlike YPet, which is pH sensitive, TOLLES evades both acid-denaturation and proteolysis inside the lysosomal lumen and retains fluorescence.

Diverse pathological conditions, including cancer, inflammatory, and neurodegenerative diseases, such as PD, have been associated with alterations in mitophagic capacity [158]. Consequently, the development of screens for compounds that modulate this fundamental cellular process holds tremendous translational potential through the discovery of novel drug targets [159]. Such screens rely on sensitive assays that measure mitophagy in both physiological and pathological conditions. Current well-established assays to monitor the selective removal of mitochondria measure different steps of the pathways described in the previous sections, and include: the quantification of endogenous or overexpressed Parkin or LC3 recruitment to the mitochondria and the quantification of the localization of mitochondria to the lysosomes compared to the cytosol [160,161]. Other methods of mitophagy detection assess mitochondrial alterations using fluorescent dyes, such as MitoTracker Deep Red or nonyl acridine orange (NAO), or by using transmission electron microscopy [162,163,164]. In addition, fluorescent reporters have been developed to measure the final steps of mitophagy, namely, the fusion of mitophagosomes with lysosomes, which we discuss in detail below (Figure 3).

### 4.1. mito-Keima

mito-Keima (mtKeima) is a pH-sensitive fluorescent biosensor that has been extensively used in a variety of cell lines, as well as in diverse model systems, including *Mus musculus* and *Drosophila melanogaster*, to measure mitophagy [52,165,166,167,168]. This reporter consists of a mutated version of the Keima protein found in stony corals, which has a pH-dependent excitation profile and a pH-insensitive emission peak at 620 nm [167,169,170]. Specifically, within a pH range of 6 to 8, which includes slightly alkali organelles, such as the mitochondria, the excitation maximum of Keima is at 440 nm [167,169,170]. By contrast, at acidic pH, which is one of the hallmarks of lysosomes, the excitation maximum of Keima shifts to 586 nm [167,169,170]. The mtKeima reporter is localized to the mitochondrial matrix by the cytochrome c oxidase subunit 8A (COX8A) targeting signal peptide sequence, artificially appended to the N-terminus of this fluorescent biosensor [167,169,170]. Consequently, mtKeima reporters found on healthy mitochondria exhibit an excitation/emission profile of 440 nm/620 nm, while those found on damaged mitochondria within autophagosomes that have fused with lysosomes have an excitation/emission profile of 586 nm/620 nm [167,169,170]. To assess the degree of mitochondrial clearance under steady-state versus pathological conditions, most often induced by protonophores such as CCCP or antimycin/oligomycin (OA), a ‘mitophagy index’ is calculated as the ratio of fluorescence intensity emitted from the two excitation peaks: 586 nm divided by 440 nm [167,169,170]. A high mitophagy index value indicates predominantly lysosomal localization of the biosensor, where it has been shown to remain remarkably insensitive to degradation by resident proteases [167,169,170]. Mitophagy index values have been obtained from different readouts, including single-cell fluorescence microscopy or flow cytometry amenable for analyzing large and diverse cell populations [167,169,170,171]. One drawback of mtKeima use is the intrinsic incompatibility of this method with fixation [159]. However, to date mtKeima has been extensively used as a robust reporter of in vivo mitophagy in different mammalian cell lines, including in induced pluripotent stem cell-derived dopaminergic neurons, and even in mice harboring a single-copy genomic integration of this reporter [52,166,167]. Notably, the latter demonstrated a remarkable degree of variability in the level of basal mitophagy among different cell types [167]. A small-scale chemical screen for modulators of mitophagy conducted with neural stem cells (NSCs) isolated from mtKeima transgenic mice identified actinonin as an inducer of this process [167]. This study demonstrated that the mtKeima mice are amenable, not only to a wide range of phenotypic studies, but also to pharmacological screens with cells isolated from diverse tissues [167].

### 4.2. mito-QC

An alternative probe to mtKeima is mito-QC. The design of this probe relies on two tandem fluorophores, mCherry and GFP, directed to the outer mitochondrial membrane by the C-terminal FIS1 transmembrane domain [172]. In the cytosol, both components of mito-QC fluoresce red and green, respectively, but when exposed to the low pH of the lysosomal compartment, the fluorescence of mCherry is maintained, while that of GFP is irreversibly lost [172]. In this case, a ‘mitophagy index’ is calculated as the count of exclusively red intracellular puncta, irrespective of size or intensity, since these puncta are thought to be resistant to lysosomal proteolysis [172]. One caveat of this approach is that mCherry and GFP have different maturation kinetics, with the former being slower than the latter, and different sensitivities to proteasomal degradation [173,174]. Unlike mtKeima, mito-QC can withstand fixation, a useful feature for colocalization with various cellular markers and analysis by fluorescence microscopy [172]. In addition to single-cell fluorescence microscopy, mito-QC has been analyzed by flow cytometry [175]. While numerous studies have used mito-QC to assay mitophagy in cell lines as well as in mice, only recently were these two reporters compared side-by-side [172,175,176,177]. The conclusions Liu et al. drew concerning the differential sensitivity level of mtKeima versus mito-QC as readouts for PINK1-Parkin-dependent mitophagy have raised debate in light of the intrinsic differences of these reporters [175,178]. Notably, systematic mito-QC analyses of basal mitophagy in different tissues isolated from *PINK1* wild type and knockout mice did not reveal remarkable differences, suggesting that PINK1 is not required for this type of mitophagy in vivo [179]. Another controversial concept in the field of mitophagy, which has been analyzed with the mito-QC and mtKeima reporters, revolves around soma-localized and axonal mitochondria. In particular, whether mitochondria in these compartments are differentially susceptible to mitophagy is still incompletely understood [180,181,182]. A sophisticated study by Harbauer et al. has begun to address this issue, demonstrating that axonal mitochondria undergo local PINK1/Parkin-mediated mitophagy [183]. Local translation of PINK1 mRNA, tethered to axonal mitochondria via Synaptojanin 2, is thought to facilitate mitophagy in distal axons by providing a supply of this labile protein [183]. Whether local translation of PINK1 mRNA occurs on mitochondrial ribosomes (mitoribosomes) remains to be directly elucidated. Nevertheless, this process circumvents the need for protein transport over long distances from the cell body, and facilitates a rapid local response to organelle damage or to bioenergetic changes within axons [184].

### 4.3. mito-SRAI

The most recently developed reporter of mitophagy is mito-SRAI. mito-SRAI consists of two tandem fluorescent proteins, acid-fast CFP or Tolerance of Lysosomal Environments (TOLLES) connected by a linker to YPet, a YFP variant [173]. The unique feature of acid-fast CFP is that it evades both acid denaturation and proteolysis inside the lysosomal lumen [173]. Consequently, acid-fast CFP fluorescence is preserved, while YPet fluorescence is lost within the lysosomes. As with the other reporters described above, mito-SRAI was extensively engineered and targeted to mitochondria not only by an N-terminal COX8A targeting signal peptide sequence, but also by C-terminal CL1 and PEST degrons that ensure the removal of free cytosolic reporters [173]. A ‘mitophagy index’ is calculated as afCFP fluorescence divided by YPet fluorescence. A high index value resulting from YPet quenching is indicative of mitophagy [173]. Similar to mito-QC, and unlike mtKeima, mito-SRAI is not sensitive to fixation [173]. Moreover, unlike the other two reporters, mito-SRAI could be used to specifically measure the mitophagy of damaged mitochondria [173]. Applying the mito-SRAI reporter to a large-scale screen of 76,000 compounds, Katayama et al. found a hit, called T-271, that effectively induced mitophagy of damaged, but not normal mitochondria, in a Parkin-dependent manner [173]. Further work is necessary to determine the molecular mechanisms involved in the selection of damaged mitochondria as opposed to healthy ones.

## 5. Future Perspectives

The shared feature of the three mitophagy assays described above is that they all report on the terminal lysosomal node of the pathway, responsible for removing damaged mitochondria. The development of robust alternatives that monitor different steps of the mitophagy pathway, amenable to high throughput studies (HTSs) will not only help advance our understanding of the mechanisms that regulate this process, but may reveal new nodes of intervention for drug targeting [159]. In particular, assays that monitor the spatiotemporal recruitment of early mitophagy effectors to the OMM in response to physiological or non-physiological stimuli, or mitophagy assays that utilize endogenous, rather than artificial fluorescent reporter systems, would open new avenues for exploration. For instance, one can envision the development of a fluorescent assay that can trace the dimerization of the BNIP3L/NIX receptor, which has been shown to be required for the induction of mitophagy [185]. By comparison with forced monomeric BNIP3L/NIX mutants, BNIP3L/NIX wild type receptors capable of forming homodimers have been shown to recruit autophagosomes more efficiently, as measured by LC3A immunofluorescence [185]. In addition, mutational analyses of key residues involved in either BNIP3L/NIX dimerization or in BNIP3L/NIX phosphorylation were demonstrated to affect mitochondrial clearance upon CCCP treatment [185]. Consequently, this dimerization event could be exploited as a mitophagy readout, potentially through a split-green fluorescent protein (GFP) system to monitor receptor homodimer formation in single cells [186,187]. This approach has been used to successfully tag members of the G-protein coupled receptor (GPCR) family of cell surface receptors [188]. Briefly, this method employs two independently non-fluorescent GFP fragments to tag a target of interest, which becomes fluorescent upon the complementation and reconstitution of a functional GFP protein [187,188]. Of note, a variation of split-GFP assays, called bi-genomic mitochondrial split-GFP, which is spatially confined to mitochondria, has recently been reported [189]. Besides mitophagy receptors, other molecules could be used as indicators of mitophagy towards assay development. For example, a recent study demonstrated that cardiolipin is externalized from the IMM to the OMM in primary cortical neurons and recruits LC3 to mitochondria, thereby initiating mitophagy [16]. This externalization process could also, in principle, be exploited as a mitophagy assay readout in a fluorescence resonance energy transfer (FRET)-based assay between a GFP-labeled probe containing the cardiolipin binding domain of the mitochondrially-localized stomatin-like protein 2 (SLP-2) and RFP-LC3 [190]. In conclusion, developing screens based on assays that rely on different steps of the mitochondrial clearance pathway holds tremendous promise for finding ways to enhance mitophagy under different pathological conditions.

## 6. Conclusions

Mounting evidence from genetic, cellular, and clinical studies over the past three decades points to the crucial role of mitochondrial dysfunction and mitophagy defects in PD. High-throughput assays, coupled with unbiased chemical or genetic screens for factors that can modulate mitophagy in susceptible dopaminergic neurons, are valuable tools for advancing PD therapies. Likewise, employing these tools to examine mitophagy in other cell types within the CNS, as well as the newly discovered process of trans-mitophagy, whereby neuron-derived damaged mitochondria are taken up and degraded by astrocytes, could offer additional points of therapeutic intervention [191,192].
